# Immunometabolic mechanisms of osteosarcopenic obesity: chronic inflammation, trained immunity, and systemic immune dysregulation

**DOI:** 10.3389/fimmu.2026.1808488

**Published:** 2026-05-07

**Authors:** Haobo Jiang, Yuting Zhang, Zicheng Luo, Xiaofei Tang, Yiyou He, Guangliang Hao

**Affiliations:** 1 Department of Orthopedics and Traumatology of Traditional Chinese Medicine, Hunan University of Chinese Medicine, Changsha, China; 2 Department of Orthopaedics, The First Affiliated Hospital of Hunan University of Chinese Medicine, Changsha, China; 3 Department of Orthopaedics, Affiliated Hospital of Shandong University of Traditional Chinese Medicine, Jinan, China

**Keywords:** Adipose-muscle-bone crosstalk, cellular senescence, chronic inflammation, immunometabolism, osteosarcopenic obesity, systems immunology, trained immunity

## Abstract

Osteosarcopenic obesity (OSO)—the co-occurrence of osteoporosis/osteopenia, sarcopenia, and excess adiposity—is increasingly recognized in ageing populations and is strongly linked to frailty, fractures, disability, and cardiometabolic complications. However, heterogeneous operational definitions and population-specific cut-offs complicate risk stratification and mechanistic inference. Here, we propose a systems immunometabolic framework to explain coordinated deterioration of adipose tissue, skeletal muscle, and bone, focusing on chronic low-grade inflammation, trained immunity (innate immune memory), and senescence-associated signaling. Dysfunctional visceral adipose tissue emerges as an immune-active endocrine organ that sustains low-grade systemic inflammation through release of cytokines, adipokines, lipotoxic mediators, and damage-associated molecular patterns. A key mechanism potentially underpinning inflammatory persistence is trained immunity—epigenetic and metabolic reprogramming of innate immune cells and their progenitors—which establishes maladaptive inflammatory memory and amplifies inter-organ immune crosstalk. In skeletal muscle, this pro-inflammatory milieu promotes catabolic signaling and anabolic resistance, including NF-κB activation and mTOR pathway dysregulation, thereby driving impaired proteostasis, fibrosis, and fatty infiltration. In bone, inflammatory and senescence-associated signals converge on osteoclastogenic pathways and disrupt the receptor activator of nuclear factor-κB ligand (RANKL)/osteoprotegerin (OPG) axis, leading to uncoupled bone remodeling and net bone loss. Collectively, we argue that OSO can be conceptualized as a fat-initiated, system-level immunometabolic remodeling process across the adipose-muscle-bone axis. This framework supports stratified, multimodal interventions combining lifestyle modification with mechanism-based anti-inflammatory and anti-resorptive therapies, while immuno-epigenetic and senescence-targeted approaches warrant further study. Notably, OSO-specific longitudinal and interventional evidence integrating immune phenotyping and multi-omics remains limited and is needed to test causality and validate actionable biomarkers and targets.

## 1 Introduction: redefining OSO as an immunometabolic disease

Osteosarcopenic Obesity (OSO) represents a devastating clinical phenotype defined not merely by coexistence of osteoporosis, sarcopenia, and obesity, but by their reciprocal and synergistic deterioration (1). It has a high prevalence among the global middle-aged and elderly population and significantly increases the risk of physical function deterioration, disability, fractures, and all-cause mortality (2, 3).

Although detrimental effects of OSO and its tendency toward younger onset have been widely recognized (2, 4), current mechanistic interpretations remain largely restricted to single-tissue senescence or isolated metabolic dysfunction, which fail to fully explain the synchronized degeneration of bone, muscle, and adipose tissue, nor the difficulty in disrupting this self-perpetuating cycle. This gap suggests that OSO arises from a system-level pathophysiological process rather than independent organ-specific decline.

We propose that OSO is fundamentally an immunometabolic disorder driven by chronic, low-grade systemic inflammation originating from dysfunctional adipose tissue and propagated through inter-organ immune and metabolic signaling pathways. Visceral adipose tissue (VAT), in particular, functions as an immune-active endocrine organ that influences distant musculoskeletal tissues through pro-inflammatory cytokines, adipokines, lipotoxic metabolites, and extracellular vesicles. These mediators generate a sustained inflammatory milieu that connects adipose inflammation with muscle catabolism and bone resorption, providing a unified explanatory model for multi-organ deterioration observed in OSO.

To further elucidate this framework, we incorporate three contemporary immunological paradigms. First, trained immunity, defined as the epigenetic and metabolic reprogramming of innate immune cells that enhances their inflammatory responsiveness upon re-stimulation, provides a mechanistic basis for the chronicity and treatment resistance of OSO (5). Second, systems immunology highlights the integrative immune network formed by cross-talk among major organs via cytokines, metabolites, and neuroendocrine circuits, which aligns with multi-organ progression characteristic of OSO (6). Third, tissue-specific Immunity emphasizes the role of microenvironment-adapted immune cells—such as adipose tissue macrophages, intramuscular macrophages, and bone marrow-resident T cells—whose polarization states critically shape local inflammatory and metabolic homeostasis.

Together, these frameworks establish a comprehensive immunometabolic perspective that helps explain chronic inflammation, multi-organ involvement, and immune dysregulation underlying OSO, and set the stage for targeted prevention and therapeutic strategies.

## 2 Adipose tissue: the engine of immunometabolic dysregulation and amplifier of inflammatory memory

### 2.1 Visceral adipose tissue inflammation as initiating event

Adipose tissue represents the primary upstream immunological driver in OSO pathogenesis, functioning not only as an energy reservoir but also as an immune-active endocrine organ that initiates and amplifies systemic immunometabolic dysfunction. In humans, adipose tissue is mainly distributed as subcutaneous adipose tissue (SAT) and VAT. Under obese conditions, VAT undergoes disproportionate expansion and becomes the dominant source of chronic low-grade inflammation (7).

VAT expansion is predominantly characterized by adipocyte hypertrophy. As adipocytes enlarge, local vascularization becomes insufficient, leading to tissue hypoxia and activation of hypoxia-inducible factor-1α (HIF-1α), which can induce adipocyte necrosis (8). In parallel, adipocyte hypertrophy is associated with excessive cellular stress and overproduction of reactive oxygen species (mtROS). These stressed or dying adipocytes release chemokines and adipokines, including monocyte chemoattractant protein-1 (MCP-1/CCL2) and leptin, which drive recruitment of circulating monocytes into adipose tissue (9). Interaction between MCP-1 and its receptor CCR2 on circulating monocytes constitutes a key pathway for macrophage recruitment (10).

In presence of free fatty acids (FFAs), lipopolysaccharide (LPS), and pro-inflammatory mediators, these infiltrating monocytes rapidly shift from homeostatic anti-inflammatory M2 phenotype toward the pro-inflammatory M1 macrophage phenotype. These macrophages subsequently organize into crown-like structures (CLSs) surrounding necrotic or stressed adipocytes (9, 11, 12). M1-polarized adipose tissue macrophages produce large amounts of tumor necrosis factor-α (TNF-α), interleukin-6 (IL-6), interleukin-1β (IL-1β), and CCL2, which spill into circulation and establish a state of persistent, low-grade systemic inflammation (13). Importantly, this inflammation is not transient; instead, it establishes a stable inflammatory tone that persists over time, even in the absence of acute metabolic insults. This persistence suggests the involvement of adaptive changes within the innate immune system itself.

### 2.2 Trained immunity: Inflammatory memory amplifying VAT-driven damage

The concept of trained immunity provides a mechanistic explanation for persistence and amplification of inflammation in OSO. Long-term exposure to high-fat and high-sugar diets, particularly those rich in saturated fatty acids, induces epigenetic and metabolic reprogramming in bone marrow hematopoietic stem cells as well as circulating innate immune cells, including macrophages and dendritic cells, through metabolic intermediates, thereby effectively “training” these cells (14, 15).

Upon stimulation by LPS, saturated fatty acids, TNF-α, and interferon-γ (IFN-γ), macrophages and dendritic cells exhibit a metabolic shift from oxidative phosphorylation (OXPHOS) to aerobic glycolysis. Even when oxygen is abundant, the cells prioritize glycolysis for rapid ATP production, while the roles of OXPHOS and fatty acid oxidation pathways are significantly diminished (16). This metabolic reprogramming sustains inflammatory response and, through intracellular fatty acid accumulation, further aggravates lipotoxicity (17, 18).

Concomitantly, activated macrophages and dendritic cells exhibit a metabolic breakpoint within the tricarboxylic acid (TCA) cycle. The intermediate citrate is exported from mitochondria and converted into acetyl-CoA. Acting as an acetyl-group donor, acetyl-CoA enhances histone acetyltransferase (HAT) activity, resulting in increased histone 3 lysine 27 acetylation (H3K27ac) at promoter regions of pro-inflammatory genes, such as TNF-α and IL-6 (19, 20). This “open” chromatin configuration facilitates transcriptional activation of these genes, even under basal conditions. Consequently, trained innate immune cells mount an amplified inflammatory response upon subsequent exposure to relatively mild metabolic stress. This form of inflammatory memory underlies the self-sustaining and self-amplifying nature of inflammation in OSO, positioning trained immunity as a critical amplifier of systemic tissue injury.

VAT, as an endocrine organ, releases adipokines, further reinforcing this phenomenon. In obesity, pro-inflammatory adipokines such as leptin and resistin are markedly elevated. In contrast, adiponectin, an anti-inflammatory and insulin-sensitizing adipokine, is substantially reduced (21). This imbalance in the pro-inflammatory/anti-inflammatory ratio is a direct mediator of systemic damage: high levels of leptin can inhibit muscle protein synthesis and promote breakdown by suppressing the IGF-Akt signal and increasing activation of the NF-κB signal. Concurrently, it activates β-2 adrenergic receptors on osteoblasts via the sympathetic nervous system, thereby reducing osteoblast differentiation and activation while enhancing osteoclast activity in bone resorption, which exacerbates bone loss (22, 23). Conversely, reduced adiponectin weakens AMPK-mediated inhibition of inflammatory signaling and exacerbates insulin resistance across systemic tissues (24).

**Figure 1:**
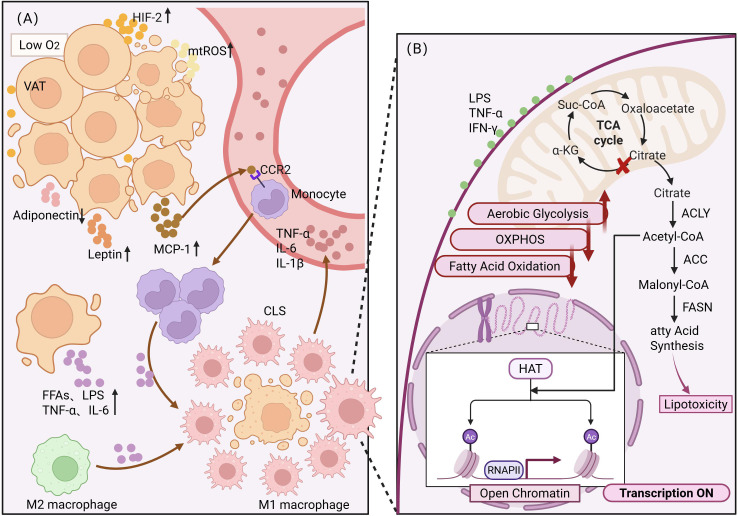
Adipose tissue-driven immunometabolic reprogramming: Systemic inflammation and self-sustaining mechanisms mediated by trained immunity. This figure illustrates how VAT in an obese state acts as the initiator of systemic inflammation, and how, through the mechanism of trained immunity in innate immune cells, inflammation is converted into a persistent inflammatory memory, ultimately driving the pathological process of OSO. **(A)** Pathological remodeling of VAT and inflammation drive. The dysfunction of hypertrophic adipocytes leads to local hypoxia and cellular stress in the microenvironment, activating HIF-1α and generating excessive mtROS. Stressed adipocytes release large amounts of chemokines, particularly MCP-1 (CCL2), attracting circulating monocytes to infiltrate the VAT. Monocytes enter the tissue by binding to the CCR2 receptor and are rapidly polarized from homeostatic anti-inflammatory M2-type macrophages to pro-inflammatory M1-type macrophages under stimulation by high concentrations of FFAs, LPS, and local inflammatory factors (TNF-α, IL-6). M1-type macrophages form CLSs around dying adipocytes and massively secrete cytokines such as TNF-α, IL-6, and IL-1β, establishing systemic chronic low-grade inflammation. Simultaneously, levels of the pro-inflammatory adipokine leptin rise, while levels of the anti-inflammatory adipokine adiponectin significantly drop, exacerbating the systemic metabolic imbalance. **(B)** Immunometabolic reprogramming and inflammatory memory in macrophages Sustained pro-inflammatory signals (LPS, TNF-α, IFN-γ, etc.) “train” innate immune cells, causing them to undergo metabolic and epigenetic reprogramming: metabolic shift: The cellular metabolic profile undergoes a Warburg effect-like shift, with enhanced aerobic glycolysis while the activity of oxidative phosphorylation and fatty acid oxidation pathways is suppressed. TCA cycle breakpoint: Citrate is transported out of the mitochondria from the TCA cycle. In the cytoplasm, ATP citrate lyase converts it into acetyl-CoA. Epigenetic memory: acetyl-CoA serves as a donor for histone acetyltransferases (HATs), enhancing histone acetylation modifications (such as H3K27ac) in promoter regions of pro-inflammatory genes, forming an “open chromatin” state. This structural change makes the genes more easily transcriptionally activated by RNA polymerase II even under basal stimulation, forming a persistent inflammatory memory. Lipotoxicity positive feedback: acetyl-CoA can also promote fatty acid synthesis via the acetyl-CoA Carboxylase and fatty acid synthase pathways, further intensifying lipotoxicity, creating a self-sustaining inflammatory vicious cycle that provides a mechanistic explanation for the refractoriness of OSO.

Collectively, these mechanisms position adipose tissue as both the engine initiating immunometabolic dysregulation and the amplifier sustaining inflammatory memory, thereby orchestrating systemic immune perturbations that drive progressive muscle wasting and bone loss in OSO. A summary of these mechanisms is shown in **Figure 1**.

## 3 Skeletal muscle: from victim of inflammation to conspirator in systemic dyshomeostasis

### 3.1 Inflammation-induced anabolic resistance and catabolic activation

In OSO, skeletal muscle is not merely a passive target of systemic inflammation but progressively transforms into an active contributor to immunometabolic dyshomeostasis. The core pathological features of skeletal muscle dysfunction in OSO include anabolic resistance, accelerated protein catabolism, and profound metabolic impairment. These alterations are primarily driven by chronic exposure to pro-inflammatory cytokines, notably TNF-α and IL-1β.

Upon binding to their respective receptors on myofibers, TNF-α and IL-1β activate the nuclear NF-κB signaling pathway. NF-κB translocate to the nucleus, upregulating the expression of key E3 ubiquitin ligase genes in the ubiquitin-proteasome system (UPS), particularly MuRF1 and Atrogin-1. MuRF1 degrades myofibrillar proteins like myosin, and Atrogin-1 degrades vimentin, thereby accelerating the net loss of muscle protein (25).

In parallel, inflammatory signaling interferes with nutrient acquisition in skeletal muscle. IL-1β-mediated NF-κB activation promotes inhibitory phosphorylation of insulin receptor substrate-1 (IRS-1), disrupting downstream phosphatidylinositol 3-kinase (PI3K)/Akt signaling. As a consequence, translocation of glucose transporter type 4 (GLUT4) to the sarcolemma is impaired, leading to impaired muscle glucose uptake capacity and skeletal muscle metabolic function (26, 27).

Mammalian Target of Rapamycin (mTOR) is crucial for muscle protein synthesis and cell growth (28). Activation of the mTORC1 signaling pathway can partially regulate skeletal muscle mass by inhibiting autophagy-mediated protein degradation (29). However, TNF-α and IL-6 can directly or indirectly inhibit the mTORC1 signaling pathway via the AMPK pathway. Inhibition of mTORC1 not only reduces translational capacity but also permits excessive autophagy and proteasomal degradation, reinforcing anabolic resistance (30, 31).

### 3.2 Disruption of muscle immune microenvironment and impaired regeneration

Skeletal muscle homeostasis depends on a finely tuned immune microenvironment that coordinates repair, regeneration, and metabolic adaptation. In healthy muscle, this niche is dominated by anti-inflammatory M2 macrophages and regulatory T cells (Tregs), which secrete cytokines such as interleukin-10 (IL-10), transforming growth factor-β (TGF-β), and amphiregulin (AREG). These signals not only suppress local inflammation, but also support activation, proliferation, and differentiation of muscle satellite cells (MuSCs), enabling efficient myofiber repair and regeneration following injury (32, 33).

In OSO, chronic systemic inflammation profoundly remodels the intramuscular immune landscape, particularly characterized by dysregulation of immunosuppressive cell populations. Persistent exposure to pro-inflammatory mediators drives macrophage polarization toward the M1 phenotype, accompanied by a marked reduction in M2 macrophages, thereby creating a hostile inflammatory milieu within muscle tissue. M1 macrophages exacerbate pro-inflammatory responses through release of pro-inflammatory cytokines, and can induce the expression of inducible nitric oxide synthase (iNOS) to promote myocyte lysis and tissue damage. Furthermore, these macrophages maintain MuSCs in a proliferative but differentiation-incompetent state, thereby impairing myogenic progression and limiting the formation of mature myofibers (32, 34). Simultaneously, high concentrations of pro-inflammatory cytokines, such as TNF-α, IL-6, and IFN-γ, not only directly inhibit the recruitment and local infiltration of Tregs, thereby reducing the abundance of Foxp3^+^ Tregs in muscle tissue, but also induce phenotypic instability in a subset of Tregs. Mechanistically, synergistic signaling mediated by IL-6, IL-23, and TGF-β drives upregulation of STAT3 and RORγt, thereby compromising Foxp3 stability (35). This drives their transdifferentiation toward a pro-inflammatory Th17-like phenotype, resulting in loss of their suppressive capacity toward effector T cells and M1 macrophages, which further impairs anti-inflammatory support required for muscle repair (33, 36). IL-17 and other pro-inflammatory cytokines secreted by Th17 cells can also induce muscle fibrosis and fatty infiltration, ultimately hindering muscle regeneration (37).

Concurrently, inflammation drives pathological expansion and dysfunction of fibro-adipogenic progenitors (FAPs), a mesenchymal cell population that normally supports muscle regeneration. Under chronic inflammatory conditions, FAPs lose their myo-supportive function and preferentially differentiate into myofibroblasts and adipocytes. This maladaptive differentiation promotes extracellular matrix deposition, muscle fibrosis, and intramuscular fat infiltration, progressively replacing contractile tissue with non-functional components (34, 38).

### 3.3 Myokine maladaptation: skeletal muscle as an endocrine contributor to systemic inflammation

Beyond its contractile role, skeletal muscle functions as a major endocrine organ through secretion of myokines that regulate systemic metabolism and immune homeostasis. In healthy states, acute exercise-induced myokine release exerts predominantly anti-inflammatory and anabolic effects. For example, short-term exercise-induced IL-6 exerts anti-inflammatory effects, enhances glucose utilization, and promotes MuSC proliferation (39, 40). Irisin, another exercise-responsive myokine, activates MuSCs and increases protein synthesis, promoting myogenesis and myoblast fusion by upregulating myogenic growth genes, thereby inducing hypertrophy and improving muscle regeneration (41). Irisin also increases the expression of Uncoupling Protein-1 (UCP-1) and stimulates the “browning” of subcutaneous white adipose tissue, enhancing insulin sensitivity and glucose uptake, thus increasing energy expenditure and ameliorating insulin resistance (42).

In OSO, the myokine secretion profile becomes profoundly dysregulated. Beneficial myokines such as irisin are reduced, whereas chronic low-level release of catabolic and pro-inflammatory factors predominates. Persistent exposure to TNF-α stimulates myostatin expression via NF-κB signaling (43). Myostatin, in turn, not only maintains muscle satellite cells in a quiescent state but also inhibits protein synthesis by stimulating proteolysis through the ubiquitin-proteasome pathway (44, 45). Elevated myostatin also promotes adipose tissue expansion, inhibits adiponectin production, and suppresses fatty acid oxidation in adipocytes, further reinforcing systemic insulin resistance (46, 47).

Chronic elevation of IL-6 in OSO differs fundamentally from its acute, exercise-induced counterpart. Sustained IL-6 signaling activates the JAK2/STAT3 pathway, leading to upregulation of suppressor of cytokine signaling 3 (SOCS3). SOCS3 interferes with insulin receptor signaling and amplifies metabolic dysfunction in skeletal muscle (48). Through these maladaptive endocrine outputs, skeletal muscle transitions from a victim of inflammation to an active contributor to systemic immune and metabolic dysregulation.

Collectively, these inflammatory and endocrine feedback loops transform skeletal muscle from a passive victim of systemic inflammation into an active amplifier of immunometabolic dysfunction. Through maladaptive immune signaling and dysregulated myokine secretion, skeletal muscle participates in a self-reinforcing pathological network linking muscle, adipose tissue, and bone, thereby accelerating the progression of OSO.

## 4 Bone: imbalance of bone remodeling driven by systemic inflammation

### 4.1 Chronic inflammation disrupts bone remodeling homeostasis

Bone remodeling is the central process by which the skeletal system maintains homeostasis, depending on the tightly coupled activities of osteoclast-mediated resorption and osteoblast-mediated formation. In OSO, this coupling is progressively lost, leading to net bone loss and increased fracture susceptibility. Accumulating evidence indicates that chronic systemic inflammation represents a central mechanism driving this pathological uncoupling.

Pro-inflammatory cytokines derived from inflamed visceral adipose tissue and skeletal muscle, including TNF-α, IL-1β, and IL-6, exert direct and indirect effects on bone cells. These cytokines directly suppress osteoblast proliferation and function while simultaneously promoting osteoclast differentiation and activation through the upregulation of NF-κB signaling, thereby accelerating the bone resorption process (49, 50). Beyond these immediate effects, they further exacerbate bone loss by perturbing the RANKL/OPG balance: primarily by upregulating RANKL expression in osteoblasts, osteocytes, and bone marrow stromal cells, which in turn activates the RANKL signaling axis, thereby promoting osteoclastogenesis; simultaneously, they reduce OPG production (51, 52). This inflammation-driven increase in the RANKL/OPG ratio disrupts bone remodeling homeostasis, leading to enhanced osteoclast activity and accelerated bone loss, ultimately underlying the fundamental pathological mechanism of increased bone resorption and elevated fracture risk in OSO.

Furthermore, the skeletal system shares the RANKL/RANK pathway with metabolic and muscular systems. This pathway is implicated in muscle metabolism, and its activation leads to a decline in skeletal muscle function and quality (53). NF-κB is a critical transcription factor downstream of the RANKL-RANK signaling pathway and is essential for mediating the inflammatory response (54). Increased ROS in myoblasts can stimulate the NF-κB pathway, thereby inhibiting myocyte proliferation and accelerating differentiation into adipocytes (55). Moreover, RANKL can promote IRS-1 Ser318 phosphorylation (56, 57), thereby interfering with insulin signaling and further exacerbating systemic metabolic dysfunction.

### 4.2 Bone marrow adiposity, cellular senescence, and SASP-mediated inflammation

Chronic systemic inflammation induces significant pathological remodeling of the bone marrow microenvironment. First, it leads to inflammatory cell infiltration, including an increase in Th17 cells. These activated T cells secrete large amounts of IL-17, which directly stimulates the expression of RANKL and further enhances bone resorption (58, 59). Second, the inflammatory milieu severely compromises the functionality of immunosuppressive subsets. Under physiological conditions, Tregs inhibit osteoclast differentiation through secretion of IL-10 and TGF-β (60). In addition, Tregs can inhibit excessive immune responses and play an important role in preventing Th17 cell activation (61). However, within the chronic inflammatory landscape, the immunosuppressive activity of bone-resident Tregs is significantly diminished; these cells may even undergo transdifferentiation into a pro-inflammatory Th17 phenotype, leading to a profound Treg/Th17 imbalance (62). Furthermore, a persistent inflammatory and RANKL-rich niche triggers metabolic and epigenetic reprogramming of myeloid-derived suppressor cells (MDSCs), facilitating their lineage commitment toward osteoclast progenitors and further exacerbating bone degeneration (63). This dysregulation of immunosuppressive cells, coupled with activation of pro-inflammatory signaling, culminates in an immune disorder within the bone marrow niche that further accelerates bone loss.

Simultaneously, inflammatory factors (e.g., TNF-α, IL-6) can activate PPARγ to induce the differentiation of bone marrow mesenchymal stem cells (BMSCs) into adipocytes rather than osteoblasts, resulting in an increase in bone marrow adipocytes (BMAs) (64, 65). Excessively expanded BMAs exert dual detrimental effects: spatially, they occupy the niches of osteoblasts and hematopoietic stem cells; via paracrine signaling, they release adipokines (e.g., leptin, resistin) and pro-inflammatory factors (e.g., TNF-α, IL-6, CCL5) to further inhibit osteoblast differentiation and promote osteoclastogenesis, thereby disrupting skeletal homeostasis (66).

The refractoriness and apparent irreversibility of OSO are largely attributable to the widespread accumulation of cellular senescence. Senescent cells accumulate in adipose tissue, skeletal muscle, and BMSCs, where they lose proliferative capacity but remain metabolically active and secrete the senescence-associated secretory phenotype (SASP) (67–69). SASP is a highly pro-inflammatory and tissue-destructive mixture, including IL-6, TNF-α, matrix metalloproteinases (MMPs), and RANKL.

SASP-driven local immune activation synergizes with systemic inflammation: for instance, IL-6 and RANKL synergistically accelerate osteoclastogenesis and inhibit osteoblast function, while MMPs degrade collagen, releasing TGF-β, which further stimulates osteoclast recruitment, thereby destroying bone homeostasis (70, 71). More destructively, SASP propagates damage to adjacent cells via extracellular vesicles (EVs) carrying microRNAs (e.g., miR-34a) and oxidized lipids (72). Concurrently, SASP factors like myostatin and TGF-β link skeletal aging with muscle atrophy by inhibiting muscle satellite cell differentiation (73). Furthermore, SASP attracts immune cells such as monocytes and neutrophils to infiltrate bone and muscle tissues, further amplifying local inflammation.

### 4.3 Bone as an active endocrine and immune modulator

Bone actively communicates with adipose tissue and skeletal muscle through the secretion of osteokines. Undercarboxylated osteocalcin (ucOCN) is a key endocrine signal among these. As a bone-derived hormone under physiological conditions, it acts directly on adipocytes to promote the secretion of adiponectin, an anti-inflammatory and insulin-sensitizing adipokine, thereby enhancing systemic insulin sensitivity. In addition, ucOCN increases energy expenditure in skeletal muscle. Osteocyte dysfunction, which impairs the secretion or carboxylation of ucOCN, weakens this protective metabolic effect, thereby exacerbating insulin resistance and inflammation in adipose tissue and increasing the metabolic burden on muscle (74).

Furthermore, osteocytes secrete sclerostin (SOST) and Fibroblast Growth Factor 23 (FGF23). SOST may promote adipogenic programming and lipid accumulation in adipocytes, whereas elevated FGF23 has been associated with metabolic dysregulation and impaired systemic insulin sensitivity. SOST stimulates pre-adipocyte differentiation by inhibiting the Wnt/β-catenin pathway and increases cellular lipid content by enhancing fatty acid synthesis and reducing catabolism (75). FGF23, primarily a regulator of phosphate metabolism, is elevated in bone-derived circulation and is closely associated with abnormal lipid metabolism in adipose tissue (76). Thus, the imbalance in the secretion pattern of these osteokines further worsens lipotoxicity in adipose tissue, ultimately solidifying the state of immunometabolic dysregulation.

Together, these processes render the skeletal component of OSO highly resistant to conventional interventions and contribute to the progressive, self-perpetuating nature of the disease.

## 5 Vicious cycle of adipose, muscle, and bone dysregulation driven by immune dyshomeostasis

**Figure 2:**
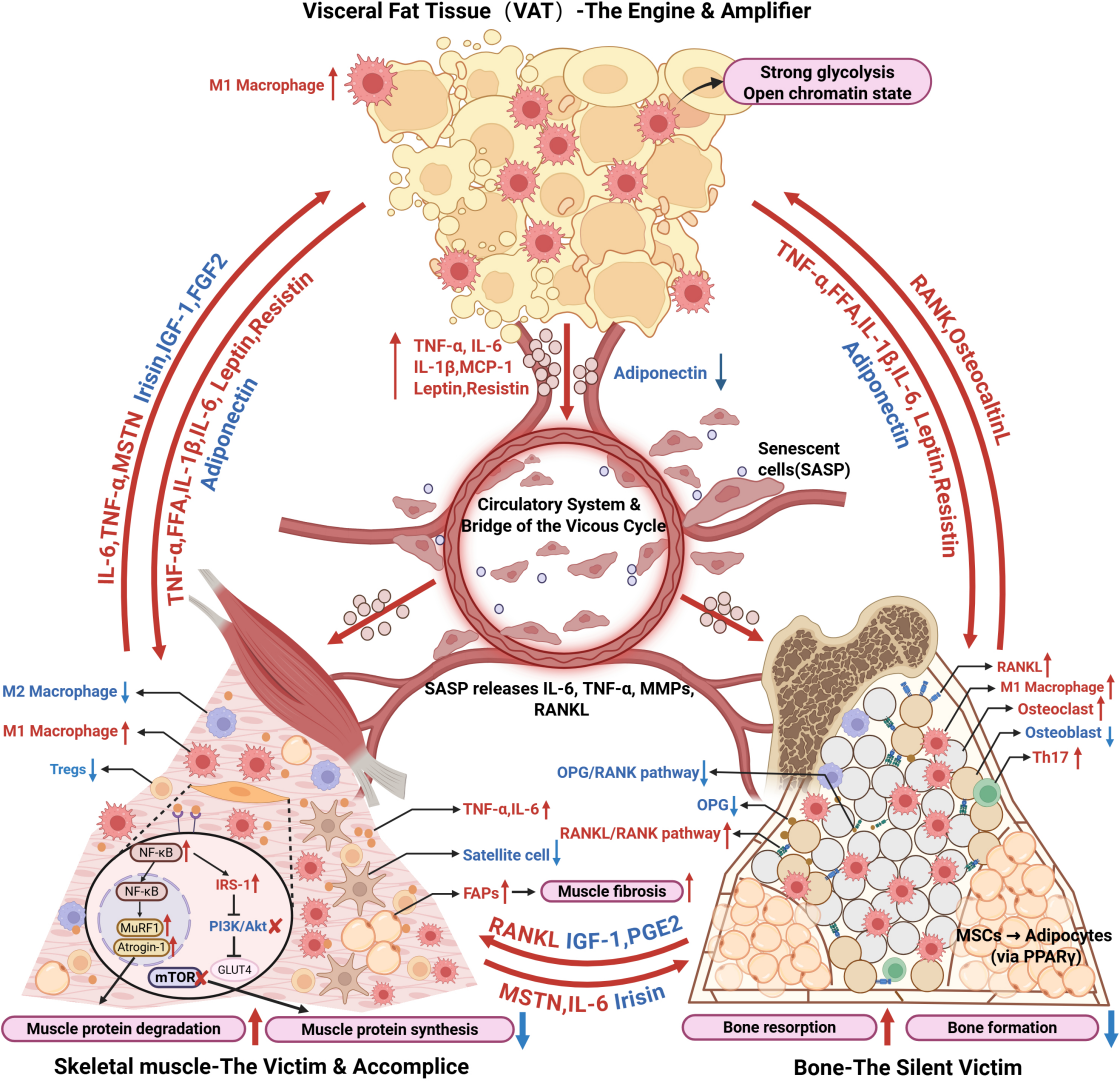
OSO is driven by a self-sustaining immunometabolic vicious cycle linking VAT, skeletal muscle, and bone. Solid red arrows or red text indicate increased mediator release, pathway activation, or pathological processes, whereas solid blue arrows or blue text denote reduced protective factors or functional inhibition. VAT acts as the primary inflammatory engine and amplifier. Adipocyte hypertrophy and immune cell infiltration promote M1 macrophage polarization and the induction of trained immunity, resulting in persistent inflammatory memory and continuous systemic release of pro-inflammatory cytokines (e.g., TNF-α and IL-6). These circulating mediators establish chronic low-grade inflammation and target skeletal muscle and bone. In skeletal muscle, inflammatory signaling activates NF-κB-dependent catabolic pathways, upregulating MuRF1 and atrogin-1, while concurrently inhibiting mTOR-mediated protein synthesis. The muscle immune microenvironment shifts toward a pro-inflammatory state, characterized by reduced M2 macrophages and regulatory T cells and increased M1 macrophages. This imbalance impairs satellite cell differentiation and promotes pathological activation of fibro-adipogenic progenitors, leading to reduced myogenesis, fibrosis, and fat infiltration. Inflamed muscle further contributes to systemic dysfunction by secreting deleterious myokines, such as myostatin. Systemic inflammation also disrupts the bone microenvironment by increasing the RANKL/OPG ratio and accelerating osteoclast-mediated bone resorption. Inflammatory stress drives bone marrow mesenchymal stem cells toward adipogenic differentiation, increasing bone marrow adiposity. Pro-inflammatory immune polarization within bone further enhances RANKL expression. Bone-derived inflammatory mediators and RANKL can enter the circulation and adversely affect muscle and adipose tissue function. Across all tissues, the accumulation of senescent cells and the associated senescence-associated secretory phenotype (SASP) reinforce immune activation and inflammatory memory, stabilizing this destructive immunometabolic loop and driving the progression of OSO.

The core pathology of OSO lies in a vicious immunometabolic cycle driven by immune dysregulation and involving a trifecta collaboration between adipose, muscle, and bone. An overview of these regulatory mechanisms is shown in **Figure 2**. This cycle is initiated by VAT-derived inflammation and further amplified by muscle- and bone-derived endocrine and immune signals that feed back to exacerbate adipose immune activation. The intricate molecular mechanisms and immunometabolic mediators involved in the pathogenesis of OSO, as well as their specific roles in tissue crosstalk, are summarized in **Table 1**. This immunometabolic vicious cycle explains the progressive, self-sustaining nature of OSO and highlights why single-organ or single-pathway interventions are insufficient. Effective therapeutic strategies must therefore target shared inflammatory and immune mechanisms across tissues to disrupt this cycle and restore systemic homeostasis.

**Table 1:** Immunometabolic mediators driving immune dysregulation in OSO.

Category	Metabolites/molecules	Primary source/ relevant tissue	Mechanisms	Primary target cells/systems	Immune dysregulation consequence	Adipose-muscle-bone axis consequence
Adipokines & adipose-derived mediators	MCP-1/CCL2	Stressed or necrotic adipocytes, M1-type macrophages	Binds CCR2 to recruit circulating monocytes into adipose tissue and enhances macrophage infiltration (9, 10).	Circulating monocytes, adipose tissue macrophages	Amplify adipose tissue inflammation and systemic low-grade inflammation.	Promotes chronic inflammatory milieu that indirectly drives muscle catabolism and bone resorption.
	Free Fatty Acids (FFAs), Lipopolysaccharide (LPS)	Adipose tissue; gut-derived endotoxin	Activate pattern-recognition receptors and promote metabolic stress; drive monocyte-to-M1 macrophage polarization in VAT (9, 11, 12).	Monocytes, adipose tissue macrophages	Favors pro-inflammatory M1 polarization and trained immunity, sustaining low-grade systemic inflammation.	Exacerbates systemic inflammation, thereby aggravating muscle wasting and bone loss.
	Leptin	Adipose tissue, bone marrow adipocytes (BMAs)	Inhibits IGF-Akt signaling and enhances NF-κB activation (22, 23); activates β-2 adrenergic receptors on osteoblasts via the sympathetic nervous system, thereby attenuating osteoblast differentiation and activation, while simultaneously promoting osteoclastogenesis (22, 23, 66).	Myofibers, osteoblasts/osteoclast lineage, circulating monocytes	Increases monocyte recruitment to VAT and promotes pro-inflammatory signaling, contributing to systemic immune activation (9).	Impairs muscle protein synthesis and accelerates muscle catabolism; disrupts bone formation and enhances bone resorption, promoting OSO progression.
	Resistin	Adipose tissue, BMAs	Promotes pro-inflammatory signaling; suppresses osteoblast differentiation and stimulates osteoclastogenesis (66).	Monocytes/macrophages, osteoblasts/osteoclast lineage	Exacerbates systemic inflammation through activation of innate immune cells and osteoimmune pathways.	Impairs bone homeostasis and contributes to bone loss and fragility; aggravates OSO-related skeletal deterioration.
	Adiponectin	Adipose tissue	Activates AMPK-mediated anti-inflammatory and insulin-sensitizing signaling (24).	Monocytes/macrophages, insulin-responsive tissues (muscle, liver, adipose)	Suppresses inflammatory activation and supports metabolic homeostasis; is markedly reduced in OSO, weakening anti-inflammatory tone.	Improved insulin sensitivity and protection against chronic inflammation under physiological conditions; its reduction in OSO aggravates systemic insulin resistance and musculoskeletal damage.
Muscle-Derived Factors	Irisin	Skeletal muscle	Promotes protein synthesis by activating myogenic stem cells (MuSCs) and upregulating myogenic growth genes, thereby inducing myogenesis and myoblast fusion (41); increases uncoupling protein-1 (UCP-1) expression to stimulate “browning” of subcutaneous white adipose tissue, improving insulin sensitivity and glucose uptake (42).	MuSCs/myofibers, adipocytes	Exerts anti-inflammatory and insulin-sensitizing effects; its levels are significantly decreased in OSO.	Promotes muscle hypertrophy and regenerative capacity; increases energy expenditure and alleviates insulin resistance; its reduction in OSO favors muscle atrophy and adipose dysfunction.
	Myostatin	Skeletal muscle	Maintains muscle satellite cells in a quiescent state and inhibits muscle satellite cell differentiation (44, 45, 73); inhibits protein synthesis by promoting proteolysis via the ubiquitin-proteasome pathway (44, 45); promotes adipose tissue expansion, suppresses adiponectin production, and inhibits fatty acid oxidation in adipocytes (46, 47).	MuSCs/myofibers, adipocytes	Fosters a catabolic and pro-inflammatory metabolic milieu and indirectly supports pro-inflammatory immune activation.	Exacerbates muscle atrophy, skeletal aging, and metabolic deterioration.
Bone-Derived Factors	Undercarboxylated osteocalcin (ucOCN)	Bone tissue	Acts directly on adipocytes to promote the secretion of the anti-inflammatory factor adiponectin; enhances insulin sensitivity; stimulates energy expenditure in myocytes (74).	Adipocytes, myocytes	Supports an anti-inflammatory, insulin-sensitizing milieu by enhancing adiponectin-linked immune-metabolic regulation; is reduced in OSO.	Its levels are decreased in OSO, exacerbating insulin resistance and inflammatory responses in adipose tissue while increasing the metabolic load on muscles.
	Sclerostin (SOST)	Osteocytes	Stimulates preadipocyte differentiation by inhibiting the Wnt/β-catenin pathway; increases cellular lipid content by promoting fatty acid synthesis and reducing catabolism (75).	Preadipocytes/adipocytes, bone microenvironment	Indirectly promotes a lipotoxic and pro-inflammatory milieu via adipogenic remodeling of bone marrow and peripheral adipose tissue.	Exacerbates bone marrow adiposity and systemic metabolic disorders, promoting lipotoxicity and contributing to bone fragility.
	Fibroblast growth factor 23 (FGF23)	Osteocytes	Elevated bone-derived levels are closely associated with abnormal lipid metabolism in adipose tissue (76).	Adipose and metabolic tissues	Associated with a pro-inflammatory, dysmetabolic state.	Exacerbates lipotoxicity and metabolic dysfunction, indirectly contributing to musculoskeletal deterioration in OSO.
Systemic Inflammatory Cytokines	TNF-α	VAT macrophages; skeletal muscle; bone marrow and other inflamed tissues	Stimulates metabolic reprogramming in macrophages and dendritic cells from oxidative phosphorylation to aerobic glycolysis (16); binds to receptors on muscle fibers to activate the nuclear NF-κB pathway, upregulating key E3 ubiquitin ligase genes in the ubiquitin-proteasome system and accelerating net muscle protein loss (25); inhibits the mTORC1 pathway directly or indirectly via the AMPK pathway (30, 31); stimulates myostatin expression through NF-κB signaling (43); induces RANKL expression while suppressing OPG production (51, 52); activates PPARγ to bias differentiation of bone marrow mesenchymal stem cells (BMSCs) toward adipocytes, increasing bone marrow adipocyte (BMA) accumulation (64, 65); suppresses muscle satellite cell differentiation (73).	T cells, macrophages, monocytes, dendritic cells, BMSCs, osteoclast precursors, myofibers	Drives M1 macrophage polarization (32, 34); impairs Treg recruitment and promotes conversion toward a Th17-like phenotype (33, 36); induces trained immunity and sustains systemic low-grade inflammation.	Establishes chronic low-grade systemic inflammation, exacerbating muscle metabolic dysfunction, sarcopenia, bone resorption, and bone marrow adiposity.
	IL-6	VAT; skeletal muscle; SASP-producing senescent cells	Acute exercise-induced IL-6: exhibits anti-inflammatory properties, enhances glucose utilization, and promotes muscle stem cell (MuSC) proliferation (39, 40). Chronic IL-6 signaling: inhibits the mTORC1 pathway via the AMPK pathway (30, 31); drives upregulation of STAT3 and RORγt, thereby compromising Foxp3 stability (35); activates the JAK2/STAT3 pathway, leading to upregulated suppressor of cytokine signaling 3, which interferes with insulin receptor signaling and exacerbates skeletal muscle metabolic dysfunction (48); promotes osteoclast differentiation and activity while inhibiting osteoblast function (64, 65); activates PPARγ to drive BMSC differentiation toward adipocytes (66).	T cells, macrophages, monocytes, dendritic cells, BMSCs, myofibers	Chronic elevation drives macrophage polarization toward the M1 phenotype (32, 34); inhibits the recruitment and local infiltration of Tregs and drive the conversion of Tregs to a Th17-like phenotype (33, 36); maintains persistent low-grade systemic inflammation.	When chronically elevated in OSO, promotes bone resorption, muscle atrophy, and skeletal muscle insulin resistance.
	IL-1β	Macrophages, inflamed tissues	Activates the nuclear NF-κB pathway to upregulate E3 ubiquitin ligase expression, accelerating muscle protein loss (25); mediates NF-κB activation to promote inhibitory phosphorylation of insulin receptor substrate-1 (IRS-1), disrupting the downstream PI3K/Akt pathway and hindering GLUT4 translocation to the sarcolemma, resulting in impaired glucose uptake and muscle metabolic dysfunction (26, 27); promotes osteoclast activity and inhibits osteoblast function (49, 50).	T cells, macrophages, monocytes, dendritic cells, BMSCs, osteoclast precursors	Drives M1 macrophage polarization; induces trained immunity and promotes IL-17 production, reinforcing pro-inflammatory immune circuits.	Sustains low-grade systemic inflammation, exacerbates muscle metabolic impairment and bone resorption, and aggravates OSO progression.
	RANKL	Osteoblasts, osteocytes, immune cells, SASP	Regulates osteoclast differentiation and activity; inhibits myocyte proliferation and accelerates differentiation into adipocytes (55); promotes IRS-1 Ser318 phosphorylation, directly interfering with insulin signaling (56, 57).	Osteoclast precursors, MDSCs, myocytes, BMSCs, T cells, myeloid cells	Induces metabolic and epigenetic reprogramming of MDSCs toward osteoclast progenitors; activates osteoimmune inflammatory pathways.	Increases bone loss and fracture risk; exacerbates muscle atrophy and systemic metabolic dysfunction.
	IL-17	Activated Th17 cells	Induces muscle fibrosis and fatty infiltration, hindering muscle regeneration (37); directly stimulates RANKL expression to exacerbate bone resorption (58, 59).	Stromal and bone cells (osteoblast lineage), myofibers/FAPs	Represents a key effector of Th17-driven inflammation, linking T cell dysregulation to bone and muscle degeneration.	Exacerbates muscle atrophy, fibrosis, and bone resorption, worsening OSO-related musculoskeletal impairment.
	TGF-β	Tregs, M2 macrophages, SASP, matrix release	Normal conditions: Promotes MuSC activation, proliferation, and differentiation for efficient myofiber regeneration after injury (32, 33); abnormal OSO conditions: drives upregulation of STAT3 and RORγt, thereby compromising Foxp3 stability (35); suppresses MuSC differentiation (73); stimulates osteoclast recruitment, disrupting bone homeostasis (70, 71).	Tregs, MuSCs, osteoclast lineage, bone microenvironment	In health, supports immune resolution; in OSO, contributes to Treg/Th17 imbalance and impaired regenerative immunity (33, 36).	Dysregulated TGF-β signaling in OSO contributes to muscle fibrosis and enhanced osteoclast recruitment.
Senescence-Associated Factors	Matrix Metalloproteinases (MMPs)	Senescent cells	Release TGF-β via collagen degradation, further stimulating osteoclast recruitment and disrupting bone homeostasis (70, 71).	Extracellular matrix, bone microenvironment	Indirectly amplify local inflammation and osteoimmune activation via matrix breakdown and growth factor release.	Promotes bone matrix destruction and amplifies local inflammation.
	miR-34a	SASP-associated extracellular vesicles (EVs)	Propagates damage signals to neighboring cells (72).	Neighboring stromal and immune cells	Sustains and spreads SASP-driven inflammatory activation and cellular dysfunction within and across tissues.	Promotes persistent inflammation, tissue degeneration, and impaired regenerative capacity in adipose, muscle, and bone compartments.

## 6 New frontiers in therapeutic strategies

### 6.1 Targeting immune inflammation

Chronic systemic inflammation is a key driver of OSO pathogenesis. Pro-inflammatory mediators, including TNF-α, IL-6, and RANKL, released by immune cells, act not only as direct effectors of bone loss and muscle atrophy but also as critical signaling hubs mediating pathological cross-talk across the adipose-muscle-bone axis. Consequently, targeting inflammatory pathways has emerged as an important strategy to restore systemic homeostasis. Current clinical and experimental studies increasingly focus on immunomodulatory agents that enable multi-tissue benefits through precise modulation of inflammatory signaling. In the following sections, we highlight the therapeutic potential of RANKL inhibitors, cytokine antagonists, JAK inhibitors, and senolytics in OSO management.

- Denosumab.
  - A fully human monoclonal antibody targeting RANKL demonstrates significant efficacy in the treatment of osteoporosis (77);
  - Blocks the binding of RANKL to RANK, inhibiting osteoclast function and increasing bone mineral density (78);
  - Improves muscle parameters and reduces fall rates in osteoporotic patients, demonstrating positive effects on both bone density and sarcopenia indicators (79);
  - Regulates the RANKL-mediated pathway to control adipocyte differentiation and indirectly affects lipid metabolism by modulating immune cell activity, particularly macrophages (80).
- TNF-α inhibitors.
  - Etanercept.
    - Promotes fracture healing by stimulating osteoblast activation through modulation of bone morphogenetic protein 2 (BMP2) and Dickkopf-1 (DKK1) signaling pathways in a rat femoral segment defect model (81);
    - Reduces weight gain induced by a high-fat diet (HFD), prevents the elevation of serum total cholesterol, triglycerides, and low-density lipoprotein cholesterol (LDL) in rats, and normalizes blood glucose levels in rats (82);
    - Has the capacity to enhance human muscle strength (83).
  - Adalimumab.
    - Prevents systemic bone loss in rheumatoid arthritis (RA) patients (84);
    - Reduces muscle wasting and alleviates cachexia by inhibiting cachexic factors like IL-6, gp130, JAK2, and STAT3 (85);
    - Reduces muscle inflammation and structural damage in mice with ischemic stroke-induced sarcopenia (ISS), increases muscle cell cross-sectional area, and improves muscle strength (86).
- Tocilizumab.
  - A humanized monoclonal antibody targeting the IL-6 receptor (IL-6R), approved for the treatment of RA refractory to methotrexate or TNF inhibitors (87).
  - Improves trabecular microstructure and tendon morphology, reduces inflammation-related bone loss, and suppresses tendon inflammation in a male rat model of collagen-induced arthritis (CIA) (88).
  - Completely reverses muscle atrophy in IL-6-overexpressing transgenic mice (89);
  - Promotes muscle fiber regeneration (90);
  - Reduces serum leptin and IL-6 levels while normalizing adiponectin levels, alleviating mild inflammatory states in rats (91).
- Mecasermin.
  - A synthetic complex of insulin-like growth factor-1 (IGF-1) and insulin-like growth factor-binding protein-3 (IGFBP-3) that improves muscle mass and metabolic function;
  - Ameliorates quadriceps muscle mass loss and reduction of muscle fiber diameter in a cisplatin-induced muscle atrophy model in mice (92);
  - Might reduce fasting insulin and abdominal fat in patients with HIV-associated lipodystrophy (93);
  - Might treat immunosenescence-associated osteoarthritis by activating the insulin-like growth factor 1 receptor (IGF1R) pathway (94).
- Tofacitinib.
  - A JAK inhibitor that primarily suppresses the JAK1 and JAK3 isoforms, thereby blocking interferon signaling and modulating immune responses (95);
  - Promotes the recruitment of BMSCs, induces osteogenic differentiation in a dose-dependent manner, lowers osteoclast activity, and slows down bone loss (96);
  - Increases muscle mass by attenuating the activation of the IL-6/JAK/STAT pathway, reducing isoproterenol expression, and restoring muscle cell differentiation markers in the tissue to baseline levels in a rabbit rheumatoid arthritis model (97);
  - Can stably confer metabolic activity on white adipocytes with similar to brown adipocytes, thereby preventing diet-induced obesity and reducing the incidence and severity of type 2 diabetes (98).
- The combination of dasatinib (D) and quercetin (Q).
  - A senolytic combination that selectively eliminates senescent cells and reduces the secretion of pro-inflammatory factors in various tissues (99).
  - Improves glucose tolerance, enhances insulin sensitivity, and lowers circulating inflammatory mediators.
  - Reduces the migration of transplanted monocytes to intra-abdominal adipose tissue, decreasing the number of macrophages in this tissue in diet-induced obese (DIO) and genetically obese mice (100).
  - Has been shown to alleviate age-related muscle loss, osteoporosis, diabetes, and obesity in experimental animals, and clinical trials are currently underway or planned (101).

A summary of these representative pharmacological agents targeting immune inflammation and their mechanisms in OSO is provided in **Table 2**.

**Table 2:** Pharmacotherapies targeting immune inflammation.

Drug name		Advantage			Limitations	References
		On bone	On muscle	On adipose tissue		
RANKL antibody	Denosumab	High-efficiency and specific binding to RANKL, thereby blocking the binding of RANKL to RANK, inhibiting the differentiation, activation, and survival of osteoclasts. This mechanism increases bone mineral density	Improves muscle parameters in patients with osteoporosis, reduces the rate of falls, and demonstrates positive effects on sarcopenia indicators.	Directly regulates the RANKL-mediated pathway, influencing adipocyte differentiation and hepatic lipid metabolism;indirectly affects lipid metabolism by modulating the activity of immune cells, especially macrophages.	Discontinuation after long-term use can lead to a rapid rebound in bone turnover markers, loss of treatment-related bone mineral density gain, and in the most severe cases, spontaneous vertebral fractures. It can also trigger safety issues such as hypocalcemia, serious infections, and osteonecrosis of the jaw.	(77–80, 101, 102)
TNF-α inhibitors	Etanercept	By regulating the BMP2 and DKK1 signaling pathways in a rat femoral segment defect model, osteoblast activation is stimulated, thereby promoting trabecular bone growth and osteocalcin expression, accelerating fracture healing.	Blocks TNF-α reduces STAT3 activation, which effectively alleviates myocardial fibrosis in rats with a high-fat diet (HFD); enhances muscle strength in healthy male volunteers.	Effectively reduces weight gain induced by HFD, prevents the elevation of serum total cholesterol, triglycerides, and low-density lipoprotein cholesterol (LDL) in rats, and restores blood glucose levels in rats to normal.	May induce dermatomyositis; in the treatment of neurodegenerative diseases such as multiple sclerosis, multiple cases of central and peripheral nervous system demyelination have been reported.	(81–83, 103, 104)
Adalimumab	Prevents systemic bone loss in patients with RA	Reduces muscle inflammation and structural damage, increases the length and weight of the soleus muscle, and increases muscle cell cross-sectional area; reduces muscle wasting and alleviate cachexia by inhibiting noxious factors like IL-6, gp130, IL-6R, JAK2, and STAT3	Inhibits cachexic factors such as IL-6, gp130, IL-6R, JAK2, and STAT3 to alleviate cachexia, resulting in less weight loss.	May lead to new onset of multiple sclerosis, pustulosis-like lesions, and lupus-like syndrome; can induce platelet antibodies leading to severe thrombocytopenia.		(84–86, 105–107)
IL-6 Receptor Antibody	Tocilizumab	Improves trabecular bone microstructure and tendon morphology, reduces inflammation-related bone loss, and suppresses tendon inflammation in the juvenile CIA rat model.	Completely reverses muscle atrophy; it has a role in promoting muscle fiber regeneration.	Reduces serum leptin and IL-6 levels while normalizing adiponectin levels alleviates mild inflammatory states in rats	Increases ectopic (visceral and cardiac) fat deposition by blocking the IL-6 receptor; infections of the skin, lungs, bones, and joints; the most common laboratory abnormalities include changes in liver function, neutropenia, and elevated cholesterol.	(88–91, 108–110)
IGF-1 Enhancer	Mecasermin	Treats osteoarthritis associated with immunosenescence by activating the IGF1R pathway.	Significantly attenuates loss of quadriceps mass and the reduction in muscle fiber diameter induced in a mouse model of cisplatin-induced muscle atrophy	Reduces fasting insulin and abdominal fat in patients with HIV infection-associated lipoatrophy	Can cause hypoglycemia (most common), lymphoid tissue hyperplasia (which may necessitate tonsillectomy/adenoidectomy), body fat accumulation	(92–94, 111, 112)
JAK Inhibitor	Tofacitinib	Promotes bone healing by recruiting BMSCs, inducing osteogenic differentiation, and reducing osteoclast activity, thereby slowing down bone loss.	Increases muscle mass by attenuating IL-6/JAK/STAT3 activation, reducing adenosine expression, and restoring muscle cell differentiation markers in the tissue to baseline levels; alleviates muscle loss in CIA mice, increases the muscle weight and muscle fiber cross-sectional area of CIA animals.	Stably confers brown fat cell-like metabolic properties to white adipocytes, preventing diet-induced obesity and reducing the incidence and severity of type 2 diabetes	May cause lymphopenia, thrombocytopenia, neutropenia, and anemia, and may even lead to serious infections, opportunistic infections, and herpes zoster.	(95–98, 113, 114)
Senolytic	D+Q	Eliminates senescent cells and alleviates osteoporosis	Eliminates senescent cells and alleviates muscle loss.	Improves glucose tolerance, enhances insulin sensitivity, reduces circulating inflammatory mediators, inhibits monocyte migration to intra-abdominal adipose tissue, and decreases the number of macrophages in this tissue.		(99–101)

### 6.2 Intervention targeting trained immunity

Epigenetic reprogramming is associated with the metabolic shift of trained macrophages, a transition that includes increased aerobic glycolysis compared to untrained macrophages. This shift depends on the activation of mTOR via the Dectin-1-AKT-HIF-1α pathway. During training, the Dectin-1 receptor mediates the phosphorylation and activation of mTOR. Activated mTOR then drives the cellular metabolic conversion from oxidative phosphorylation to aerobic glycolysis by upregulating the transcription factor HIF-1α, which is accompanied by epigenetic reprogramming, such as histone modifications, enabling cells to acquire enhanced immune responsiveness. Rapamycin (sirolimus) is a common mTOR inhibitor that has been shown to specifically inhibit mTOR signaling, preventing the activation of HIF-1α, thereby affecting the glycolytic conversion and weakening the induction of trained immunity (115). Therefore, rapamycin shows considerable potential in treating musculoskeletal metabolic diseases associated with OSO. Rapamycin restores normal autophagic flux in skeletal muscle cells in mice by inhibiting mTORC1 activity, thereby restoring muscle function (116). Luo et al. (117) administered rapamycin to 24-month-old rats and found increased trabecular bone mineralization, accompanied by a decrease in osteoclasts and enhanced osteocyte autophagy. Additionally, studies show that rapamycin can reduce inflammation by inhibiting the protein translation of membrane-bound IL-1α and suppressing the transcription of many genes encoding SASP factors (118).

In clinical practice, epigenetic drugs are generally classified into histone deacetylase (HDAC) inhibitors, DNA methyltransferase (DNMT) inhibitors, and histone methyltransferase inhibitors. These inhibitors influence chromatin structure by targeting the reversible modifications involved in DNA or histones to achieve phenotype alteration and disease treatment (119). Among these, vorinostat, a canonical HDAC inhibitor, and decitabine, a representative DNMT inhibitor, have recently demonstrated significant multitargeted therapeutic potential in modulating skeletal muscle atrophy, inhibiting osteoclast activity, and ameliorating lipid metabolism dysregulation.

Studies have shown that vorinostat promotes muscle differentiation indirectly by increasing histone acetylation levels and reducing adipogenesis, helping to protect mouse muscle from degeneration after rotator cuff (RC) injury (120). Based on transcriptomic analysis, vorinostat is a promising candidate drug for the treatment of sarcopenia (121). A 2024 experiment found that vorinostat exhibits significant anti-bone loss properties by regulating RANKL-induced signaling to inhibit osteoclast bone resorption in ovariectomized mice (122). Furthermore, vorinostat can inhibit *in vitro* human osteoclast bone resorption at low nanomolar concentrations (123).

Decitabine, in turn, achieves epi-therapy by regulating aberrant DNA methylation patterns in a reversible manner through inhibiting DNMT activity (124). In recent years, studies have shown that decitabine reduced hepatic lipid accumulation and increased lipid content in skeletal muscle in mice fed a high-animal fat and high-protein diet, promoting mitochondrial quality control and function, and exhibiting a metabolic memory effect. Decitabine can also inhibit osteoclast-specific genes (including RANK) and signaling pathways (such as NF-κB, AP-1, and ERK), effectively preventing bone loss and suppressing osteoclastogenesis (125). Decitabine has not been widely applied in OSO, but its aforementioned functions suggest its potential as a drug for OSO (**Table 3**).

**Table 3:** Intervention of trained immunity for OSO treatment.

Drug name		Advantage			Limitations	References
		On bone	On muscle	On adipose tissue		
mTOR inhibitor	Rapamycin (Sirolimus)	Reduces the number of osteoclasts and osteocyte apoptosis, and alleviates age-related skeletal changes by activating osteocyte autophagy.	Inhibits mTORC1 activity in mice, restores skeletal muscle cell autophagy flux to normal, enabling muscle recovery; alleviates age-related muscle atrophy.	Inhibits the protein translation of membrane-bound IL-1α, suppresses the transcription of numerous genes encoding SASP factors, and thereby reduces the occurrence of inflammation.	It can induce weight loss in patients, reduce glucose uptake and oxidation in skeletal muscle, leading to cachexia and muscular atrophy; long-term use can result in significant loss of muscle mass.	(115–118, 126–128)
Epigenetic drugs	Vorinostat	Inhibits osteoclast-mediated bone resorption in ovariectomized mice by regulating RANKL-induced signaling pathways, exhibiting prominent anti-bone loss activity; suppresses bone resorption in cultured human osteoclasts at low nanomolar concentrations.	Elevates histone acetylation levels, indirectly promotes myogenic differentiation, and prevents muscle degeneration in mice with rotator cuff injury; reduces the degeneration of spinal motor neurons in a mouse model of spinal muscular atrophy, enhances neuromuscular junction formation, and increases myofiber size, thereby significantly improving motor function in mice.	Increases histone acetylation levels and reduces adipogenesis	Side effects including gastrointestinal symptoms, fatigue, thrombocytopenia, and even thrombosis	(120–122, 129, 130)
	Decitabine	Inhibits osteoclast-specific genes (including RANK) and signaling pathways (e.g., NF-κB, AP-1, and ERK), effectively preventing bone loss and suppressing osteoclastogenesis.	Increases lipid content in the skeletal muscle of mice on a high-protein diet, promotes mitochondrial quality control and function, and exhibits the effect of metabolic memory.	Reduces hepatic lipid accumulation in mice fed a diet high in animal fat and protein.	Continuous administration may lead to mild to moderate neutropenia, thrombocytopenia, or other infections.	(124, 125, 131)

### 6.3 Lifestyle interventions

Currently, there is no definitive treatment strategy for OSO, but relevant research indicates that exercise is an effective intervention that can, to some extent, improve clinical symptoms. Resistance training (RT), an exercise program designed to enhance strength, has been widely used in the treatment of sarcopenia or sarcopenic obesity (132, 133). Numerous studies have shown that RT is also an effective strategy for improving OSO (134–136). By increasing beneficial myokines (such as IL-6, IL-10, IGF-1, and irisin) in muscle, while decreasing myostatin, FOXO, leptin, and resistin, RT can promote muscle protein synthesis, reduce adipose tissue and pro-inflammatory factors, and alleviate the burden of SASP. Specifically, IL-10, as a powerful anti-inflammatory cytokine, directly inhibits macrophage M1 polarization and the release of pro-inflammatory factors like TNF-α and IL-2, improving the pro-inflammatory microenvironment of muscle (137). A meta-analysis by Yang et al. (134) showed that 12 weeks of elastic band resistance training effectively increased bone mineral density and significantly improved skeletal muscle mass, while markedly reducing body fat percentage in elderly patients with OSO. Moreover, the combination with moderate-intensity aerobic exercise showed better results (138). Aerobic exercise can elevate the peak oxygen consumption and basal metabolic rate of OSO patients and significantly reduce body fat (139). However, current research on the effects of aerobic exercise or combined training on OSO patients remains limited, and most included intervention studies have a relatively short duration (primarily 12 weeks), which may be insufficient to induce significant changes in skeletal muscle mass and inflammatory markers; therefore, further research is required.

Some exercise studies report that high-intensity resistance training is considered the gold standard intervention. However, in clinical settings, high-intensity exercise is not always feasible for elderly and frail patients (140). Some scholars have found that low-intensity resistance training combined with blood flow restriction (BFR) exercise has emerged as an innovative intervention (141). BFR training restricts venous blood return from the limbs while maintaining arterial blood flow, leading to local ischemia and hypoxia in the distal muscles. In this environment, despite the low external load, local metabolic stress and lactic acid accumulation rapidly increase, potently activating fast-twitch fibers, recruiting motor units, and stimulating muscle protein synthesis pathways such as mTORC1, thus achieving synthetic effects similar to high-intensity RT under low mechanical load (142). BFR can also effectively upregulate anabolic factors (such as IGF-1), inhibit FOXO activity, and improve the muscle microenvironment, thereby effectively reversing trained immunity and mitigating the SASP burden (141).

Nutritional therapy is also an effective strategy for treating OSO (143). The Mediterranean diet has garnered widespread attention in recent years. While there is currently no explicit research on the efficacy of the Mediterranean diet for OSO, there is substantial evidence supporting its use in treating osteoporosis, sarcopenia, and obesity, respectively. The Mediterranean diet is a healthy eating pattern characterized by high consumption of fruits, vegetables, whole grains, and fish, and it is rich in anti-inflammatory nutrients such as fiber, omega-3 fatty acids, monounsaturated fatty acids, and olive oil polyphenols (144). Olive oil, a key component of the Mediterranean diet, has been shown to protect bone health and slow muscle loss through its anti-inflammatory effects and reduction of oxidative stress (145, 146). Meta-analyses of multiple studies have shown that the Mediterranean diet can significantly reduce pro-inflammatory cytokines in the body, such as IL-6, IFN-γ, TNF-α, and CRP, and increase anti-inflammatory factors IL-10 and IL-15, greatly improving adipose tissue inflammatory environment and lowering the risk of obesity (147, 148). Phytochemicals in the Mediterranean diet (such as carotenoids and vitamins C and E) possess antioxidant properties that reduce cellular stress and DNA damage. This not only aids in muscle anabolism and improved bone density but also indirectly acts as a senescence modulator by reducing the inflammatory burden and oxidative stress, inhibiting SASP secretion of senescent cells, thereby synergistically mitigating the pathophysiological progression of OSO syndrome (149–152).

Beyond dietary patterns, selected nutrient-based interventions may modulate OSO through more specific endocrine, anti-inflammatory, and immunometabolic mechanisms. Deficiency of vitamin D, a fat-soluble steroid hormone, is closely linked to the pathogenesis of OSO (153). It not only promotes calcium absorption by inhibiting parathyroid hormone (PTH) levels to reduce the risk of falls and fractures, but also directly stimulates muscle cell proliferation and growth by activating the Vitamin D receptor (VDR). In adipose tissue, Vitamin D demonstrates the capacity to influence energy metabolism and potentially modulate adipogenesis and pre-adipocyte differentiation through VDR regulation (154). Clinical evidence further highlights that synergistic regimens, combining Vitamin D with protein or amino acid supplementation, or whole-body vibration training, can enhance bone mineral density, muscle mass, strength, and physical function by regulating myokines such as irisin and MSTN (155). The synergistic treatment of OSO with Vitamin D and other nutrients is a key direction for future exploration.

Similarly, dietary-derived bioactive polysaccharides, such as β-glucans, modulate OSO-related pathological processes through potent anti-inflammatory and metabolic regulatory effects. β-glucans are a class of bioactive fibers or polysaccharides proven to exert positive effects on musculoskeletal health and effectively mitigate obesity (156). For instance, use of β-glucans reduced RANK-L expression in animals with periodontal disease, inhibited osteoclast formation, and promoted their apoptosis, thereby alleviating bone loss (157). Experiments by Zhang et al. showed that oat β-glucan treatment reversed TNF-α-induced abnormal myoblast differentiation and reduced expression levels of MuRF-1 and Atrogin-1, mitigating muscle atrophy (156). β-glucan intake can also improve high blood sugar levels and insulin resistance, demonstrating anti-obesity and beneficial metabolic effects (158). However, systematic studies on the clinical and physiological significance of β-glucan are still scarce, and whether it can become a therapeutic agent for OSO awaits future research.

**Figure 3:**
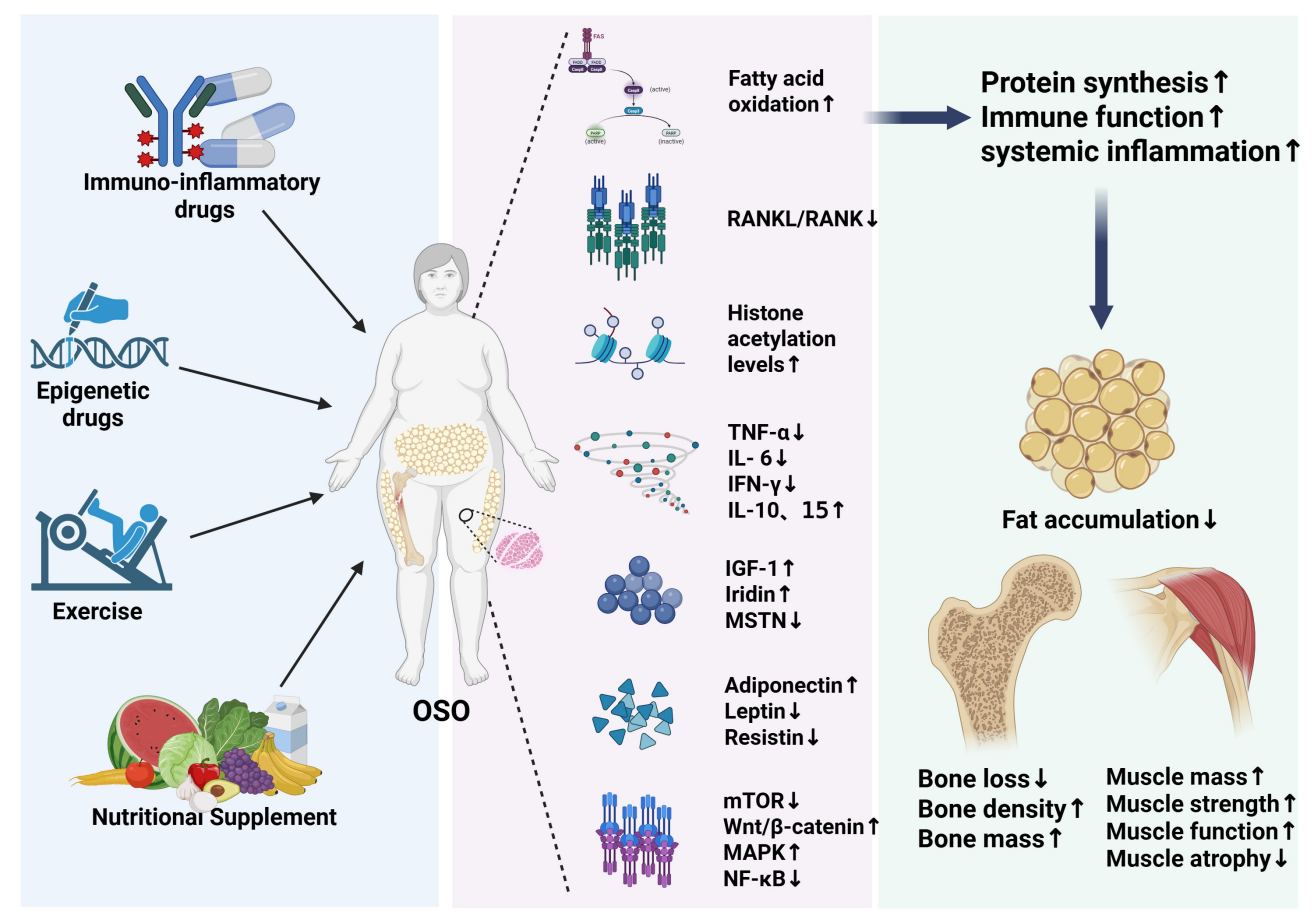
The figure illustrates the regulatory mechanisms and effects of immuno-inflammatory drugs, epigenetic drugs, exercise, and nutritional supplements on OSO. The left side presents the interventions, the middle shows their regulation at the molecular level of metabolism, epigenetics, and cytokines (e.g., enhanced fatty acid oxidation, downregulation of RANK/RANKL, modulation of changes in inflammatory factors like TNF-α and IL-6, changes in adipokines like adiponectin, changes in myokines like irisin, and regulation of signaling pathways like mTOR). The right side reflects the ultimate physiological effects: increased protein synthesis, improved immune function, coupled with reduced fat accumulation, alleviated bone loss, and enhanced muscle mass and function. This figure intuitively presents the positive regulatory network of multi-dimensional interventions on the musculoskeletal metabolism and systemic status in OSO.

It is worth noting that exercise and nutritional therapy enhance muscle strength and prevent bone loss more effectively than single treatments. Studies indicate that protein and Vitamin D supplements combined with resistance training help maintain muscle mass and bone density while reducing fat accumulation, effectively slowing the progression of OSO (159). A study in the United States showed that after 6 months of a low-fat, vitamin-rich, balanced diet, and exercise intervention (endurance training and strength training), inflammatory factors CRP, IL-6, and body weight were significantly reduced in older adults (>65 years), with improvements also seen in insulin sensitivity and blood lipid levels (160). Therefore, the role of lifestyle interventions involving exercise and nutritional therapy should be emphasized in the prevention and treatment of OSO. A summary of these treatment strategies can be found in **Figure 3** and **Table 4**.

**Table 4:** Lifestyle Interventions for treatment.

Intervention method	Advantage			Limitations	References
	On bone	On muscle	On adipose tissue		
Resistance exercise	Increases bone mineral density.	Increases beneficial myokines in muscle, including IL-6, IL-10, IL-15, IGF-1, irisin, FGF-21, LIF, and adiponectin, while reducing myostatin, FOXO, leptin and resistin, thereby promoting muscle protein synthesis and increasing muscle mass.	Reduces adipose tissue and pro-inflammatory factors, leading to decreased fat mass.	Currently, research on the effects of aerobic exercise or combined training in patients with OSO remains limited, and most of the included studies have a relatively short intervention duration (primarily 12 weeks).	(134, 137)
Mediterranean diet	Improves bone mineral density by exerting anti-inflammatory effects and reducing oxidative stress.	Helps with muscle anabolism, alleviate oxidative stress and inflammatory burden, and reduce the risk of muscular atrophy.	Significantly decreases the levels of pro-inflammatory cytokines such as IL-6, IFN-γ, TNF-α, and CRP in the body, while increasing the levels of anti-inflammatory cytokines including IL-10 and IL-15, thereby substantially ameliorating the inflammatory microenvironment of adipose tissue and reducing the risk of obesity.	Combined exercise and nutritional intervention exerts more pronounced effects on enhancing muscle strength and preventing bone loss than monotherapy.	(147–149– 152, 161, 162)
Vitamin D	Inhibits parathyroid hormone (PTH) levels, promotes calcium absorption, and reduces the risk of falls and fractures.	Activates the Vitamin D receptor (VDR) to stimulate the proliferation and growth of muscle cells.	Modulates VDR to affect energy metabolism, and may regulate adipogenesis and preadipocyte differentiation.	The therapeutic effects are not significant enough to verify the causal relationship between OSO and Vitamin D deficiency.	(153, 154)
β-glucan	Reduces RANK-L expression in animals with periodontal disease, inhibits osteoclastogenesis and promotes osteoclast apoptosis, thereby alleviating bone loss; stimulates the proliferation, differentiation and mineralization potential of osteoblasts via the BMP-2, Wnt/β-catenin and MAPK signaling pathways, exerting a therapeutic effect on osteoporosis	Reverses TNF-α-induced abnormal myoblast differentiation, decreases the expression levels of MuRF-1 and Atrogin-1, and alleviates muscular atrophy.	Improves hyperglycemia and insulin resistance, exhibiting anti-obesity activity.	Systematic studies on the clinical and physiological significance of β-glucan are still scarce, and whether it can become a therapeutic agent for OSO awaits future research.	(156–158, 163)

Taken together, these interventions target complementary nodes across the adipose-muscle-bone axis; their mechanisms, evidence base, and translational limitations are summarized in **Table 5**.

**Table 5:** Summary table of therapeutic strategies: multi-dimensional interventions for OSO.

Category	Intervention	Targets/mechanisms	Effects on the adipose-muscle-bone axis	Research stage
Targeting immune inflammation	Denosumab	RANKL inhibition	↑BMD; ↑muscle parameters; regulation of lipid metabolism	Clinical application/Animal studies
	Etanercept	TNF-α blockade	↓Bone loss; ↑muscle strength; ↓serum lipids and blood glucose	Clinical application/Animal studies
	Adalimumab	TNF-α blockade	↓Bone loss; ↑muscle strength; ↓muscle atrophy; ↓local inflammation	Clinical application/Animal studies
	Tocilizumab	IL-6R blockade	↑Bone microstructure; ↑muscle fiber regeneration; ↓muscle atrophy; ↓inflammation	Clinical application/Animal studies
	Mecasermin	IGF-1 enhancement	↓Osteoarthritis; ↓muscle atrophy; ↓fat mass	Clinical application/Animal studies
	Tofacitinib	Inhibition of JAK/STAT pathways	↑Osteogenesis; ↓bone loss; ↑muscle mass; ↓obesity	Animal studies
	Dasatinib + Quercetin (D+Q)	Clearance of senescent cells (senolytics)	↑Glucose tolerance; ↑insulin sensitivity; ↓inflammatory mediators	Clinical application/Animal studies
Intervention of trained immunity	Rapamycin	mTOR inhibition	↑Bone mineralization; ↑muscle function; ↓inflammatory response	Animal studies
	Vorinostat	HDAC inhibition	↓Bone loss; ↑muscle differentiation	Animal studies
	Decitabine	DNMT inhibition	↓Osteoclastogenesis; ↓bone loss; ↑muscle metabolism; ↓lipid accumulation	Animal studies
Lifestyle interventions for treatment	Resistance Exercise	Myokine regulation	↑BMD; ↑protein synthesis; ↑muscle mass; ↓body fat	Clinical application/Animal studies
	Mediterranean Diet	Anti-inflammatory; antioxidant	↑BMD; ↑muscle anabolism; ↓muscle loss; ↓obesity risk	Clinical application/Animal studies
	Vitamin D	PTH inhibition; VDR activation	↑BMD; ↑muscle mass; ↑muscle strength; ↓adipogenesis	Clinical application/Animal studies
	β-glucan	Anti-inflammatory; metabolic regulation	↓Bone loss; ↓muscle atrophy; anti-obesity and hypoglycemic effects	Animal studies

### 6.4 Limitations of current interventions and potential treatment strategies

Despite the multidimensional therapeutic potential demonstrated by pharmacological, exercise, and nutritional interventions for OSO, significant challenges remain in the clinical application. Immunomodulatory drugs are associated with substantial risks of adverse effects. For instance, while RANKL antibodies, TNF-α inhibitors, and JAK inhibitors effectively suppress inflammatory responses, long-term administration may increase the risk of infection and impair normal immune surveillance (101, 105, 108, 113); furthermore, their efficacy across distinct tissues of the adipose-muscle-bone axis exhibits marked heterogeneity. Additionally, most studies remain rooted in single-disease models, such as rheumatoid arthritis or osteoporosis, lacking high-quality clinical evidence specifically tailored to OSO as a multisystem syndrome. Epigenetic drugs targeting trained immunity also present non-negligible adverse reactions; clinical observations indicate that patients treated with decitabine and vorinostat frequently experience thrombocytopenia and other infectious events, casting doubt on their long-term safety (129–131). Moreover, epigenetic regulation and trained immunity interventions are still in early exploratory stages. Although mTOR inhibitors and HDAC/DNMT inhibitors show potential in modulating inflammation and metabolic reprogramming in experimental settings, their long-term safety, dose-dependent effects, and tissue-specific mechanisms remain poorly understood, limiting their broad application. Lifestyle interventions, while the cornerstone of OSO prevention and management, are often constrained by poor long-term patient adherence and represent a significant physiological burden for the elderly and frail individuals. Furthermore, exercise alone cannot eradicate the inflammatory memory established by trained immunity; once the intervention ceases, patients are highly susceptible to reverting to a pathological state. Similarly, while nutritional supplements possess certain regulatory capacities, their clinical efficacy remains modest, precluding them from being a primary therapeutic strategy. Most lifestyle studies involve short-term interventions and lack long-term follow-up data to evaluate sustained impacts on bone mineral density, muscle mass, and fat distribution.

To address the aforementioned limitations of these therapies, particularly their lack of tissue specificity and potential for systemic adverse effects, the development of advanced biomaterials represents a highly promising, albeit still emerging, frontier. Although OSO-specific biomaterial-based therapies have not yet been established and direct clinical evidence remains lacking, these technologies offer a potential strategic platform for the precision treatment of OSO. Intelligent drug delivery systems, constructed using bone-targeting nanoparticles, exosomes, or responsive-release materials, can precisely deliver anti-inflammatory drugs or growth factors to damaged bone microenvironments or atrophied muscle tissues. This enhances local bioavailability while minimizing systemic toxicity (164). Furthermore, immunomodulatory tissue engineering scaffolds—such as bioceramic composites or multifunctional hydrogels—can mimic the extracellular matrix to provide mechanical support for damaged tissues. These materials promote synergistic tissue regeneration by locally releasing bioactive molecules that reverse the microenvironmental deterioration (165).

Photobiomodulation (PBM), as a non-invasive physical therapy that does not rely on drugs or biomaterials, provides an alternative strategy to traditional pharmacological interventions (166). Experimental evidence confirms that PBM, utilizing low-energy red and near-infrared light, not only significantly improves the morphology and function of muscle fibers while reducing local oxidative stress and inflammation, but also enhances osteogenic differentiation to promote bone repair (167, 168). Additionally, PBM can decrease the levels of adipocyte differentiation markers and the expression of inflammatory cytokines (169, 170). Although comprehensive studies directly applying PBM to OSO are currently lacking, its profile as a physical therapy with minimal side effects suggests it may open new avenues for OSO treatment.

Future OSO therapeutic strategies should focus on the deep integration of combination therapies and personalized precision medicine. Establishing comprehensive regimens—such as biomaterial carriers + pharmacotherapy + exercise + nutritional support—holds promise for achieving synergistic sustained release and long-term regulation of multiple active components. Furthermore, novel intervention strategies targeting senescent cells and immunometabolic abnormalities—such as highly selective senolytics, metabolic reprogramming regulators, and gut microbiota modulators—are likely to become key research priorities. In summary, while current OSO treatment strategies have made progress, multidisciplinary cross-research and high-quality clinical trials are essential to drive the transition from single-target interventions toward systemic precision regulation, ensuring long-term effective management of OSO.

## 7 Discussion

This review proposes an integrated immunometabolic perspective on OSO. Rather than representing a simple coexistence of osteoporosis, sarcopenia, and obesity, OSO may be conceptualized as a adipose tissue-initiated, systemic immunometabolic remodeling process, characterized by chronic low-grade inflammation, maladaptive innate immune programming (including trained immunity/innate immune memory), and progressive musculoskeletal dysfunction. Importantly, heterogeneous operational definitions and cut-offs remain a major barrier to cross-cohort comparability and causal inference, and OSO may not yet meet criteria for a discrete clinical entity in all settings.

A working model is that OSO involves a self-perpetuating pathogenic network. Pathologically expanded and remodeled VAT functions as a primary inflammatory hub, continuously releasing adipokines, cytokines, and danger-associated molecular patterns that initiate and sustain systemic immune activation. Concurrently, trained immunity—long-term functional reprogramming of innate immune cells and their progenitors mediated by epigenetic and metabolic changes—may contribute to persistent inflammatory signaling and help explain the chronicity and partial refractoriness observed in some OSO trajectories. Superimposed on this process, the accumulation of senescent cells and their SASP further amplifies inflammatory signaling. Together, these processes converge on key immunometabolic pathways, including the NF-κB/mTOR and RANKL/OPG axes, leading to coordinated deterioration of skeletal muscle and bone and, ultimately, functional collapse of the adipose-muscle-bone axis.

It is imperative to acknowledge the existing controversies and evidence boundaries that define the current understanding of OSO. First, the directionality and relative contribution of adipose-derived versus muscle- and bone-derived endocrine/immune feedback loops likely vary across sex, age, adiposity distribution, comorbidities, and medications. Second, much of the mechanistic evidence for trained immunity and senescence-driven amplification is extrapolated from obesity and cardiometabolic disease contexts; direct OSO-specific longitudinal and interventional evidence remains limited, and reverse causality and confounding cannot be excluded. Third, the field needs consensus diagnostic criteria with clinically relevant cut-offs to enable robust stratification and reproducibility. Therefore, these mechanisms may represent causal drivers, downstream adaptations, or both, depending on disease stage and tissue context.

Future research paradigms should shift from single-organ symptom management toward systems-level, network-targeted hypotheses. The primary scientific challenge is to map the high-resolution inter-organ immune crosstalk, particularly clarifying how signaling molecules originating from adipose tissue precisely target the bone marrow microenvironment and induce epigenetic reprogramming in hematopoietic stem/progenitor cells. Furthermore, dissecting the dynamic interaction between tissue-specific microenvironments (such as immune cell subsets in intramuscular fat infiltration areas) and the systemic immune status will provide critical clues for identifying new therapeutic targets.

From a translational perspective, the management of OSO is likely to require a stratified, multimodal intervention strategy. Lifestyle-based interventions, including anti-inflammatory dietary patterns and combined resistance and aerobic exercise, should form the foundation of OSO prevention and treatment, given their broad immunometabolic regulatory effects. Pharmacological agents targeting key inflammatory and metabolic pathways—such as RANKL- and IL-6-related signaling—may provide additional benefit in selected patient populations by interrupting pathological inter-organ communication. Finally, emerging strategies aimed at modulating epigenetic programs underlying trained immunity and reducing senescent cell burden represent a promising, though still exploratory, therapeutic frontier.

In summary, integrating lifestyle interventions with precision therapies targeting immunometabolic dysregulation offers a conceptual shift in OSO management—from symptomatic control toward mechanism-informed, potentially disease-modifying strategies. By explicitly addressing definitional heterogeneity, evidence limitations, and testable systems-level hypotheses, this framework provides a rational foundation for future clinical and translational research.
